# The economic burden of prematurity in Canada

**DOI:** 10.1186/1471-2431-14-93

**Published:** 2014-04-05

**Authors:** Karissa M Johnston, Katherine Gooch, Ellen Korol, Pamela Vo, Oghenowede Eyawo, Pamela Bradt, Adrian Levy

**Affiliations:** 1 Epidemiology, Oxford Outcomes Ltd., Vancouver, Canada; 2 Abbvie Inc, North Chicago, IL, USA; 3 Faculty of Health Sciences, Simon Fraser University, Burnaby, Canada; 4 Adzoe Inc., Libertyville, USA; 5 Department of Community Health & Epidemiology, Dalhousie University, 5790 University Ave., Halifax, Nova Scotia B3H 1V7, Canada

## Abstract

**Background:**

Preterm birth is a major risk factor for morbidity and mortality among infants worldwide, and imposes considerable burden on health, education and social services, as well as on families and caregivers. Morbidity and mortality resulting from preterm birth is highest among early (< 28 weeks gestational age) and moderate (28–32 weeks) preterm infants, relative to late preterm infants (33–36 weeks). However, substantial societal burden is associated with late prematurity due to the larger number of late preterm infants relative to early and moderate preterm infants.

**Methods:**

The aim in this study was to characterize the burden of premature birth in Canada for early, moderate, and late premature infants, including resource utilization, direct medical costs, parental out-of-pocket costs, education costs, and mortality, using a validated and published decision model from the UK, and adapting it to a Canadian setting based on analysis of administrative, population-based data from Québec.

**Results:**

Two-year survival was estimated at 56.0% for early preterm infants, 92.8% for moderate preterm infants, and 98.4% for late preterm infants. Per infant resource utilization consistently decreased with age. For moderately preterm infants, hospital days ranged from 1.6 at age two to 0.09 at age ten. Cost per infant over the first ten years of life was estimated to be $67,467 for early preterm infants, $52,796 for moderate preterm infants, and $10,010 for late preterm infants. Based on population sizes this corresponds to total national costs of $123.3 million for early preterm infants, $255.6 million for moderate preterm infants, $208.2 million for late preterm infants, and $587.1 million for all infants.

**Conclusion:**

Premature birth results in significant infant morbidity, mortality, healthcare utilization and costs in Canada. A comprehensive decision-model based on analysis of a Canadian population-based administrative data source suggested that the greatest national-level burden is associated with moderate preterm infants due to both a large cost per infant and population size while the highest individual-level burden is in early preterm infants and the largest total population size is in late preterm infants. Although the highest medical costs are incurred during the neonatal period, greater resource utilization and costs extend into childhood.

## Background

Preterm birth, defined as birth before the completion of 37 weeks gestation, [1] is a major risk factor for morbidity and mortality among infants worldwide, and imposes considerable burden on health, education and social services, as well as on families and caregivers [1-5]. The epidemiologic burden of prematurity in Canada is substantial; approximately eight percent of live in-hospital births in 2009–2010 were preterm; [6,7] and considerably high hospital costs and other health expenditures have been reported for this population.

Morbidity and mortality resulting from preterm birth is highest among early (born at less than 28 weeks gestational age) and moderate (born between 28 and 32 weeks gestational age) preterm infants [8,9]. The morbidity impact of preterm birth is not limited to the neonatal period, but also extends into later periods in life resulting in cognitive developmental impairments, learning difficulties, social and behavioral problems [8,10,11]. Learning disability is associated with considerable costs to individuals, families, and the society [12]. The epidemiology, causes and outcomes of preterm birth have been extensively reviewed [2,8,10,13]. Due to the underdeveloped lung tissue, respiratory morbidity is commonly associated with prematurity. Less common prematurity-associated morbidities include sepsis, intraventricular hemorrhage, periventricular leukomalacia, necrotizing enterocolitis, cerebral palsy, retinopathy of prematurity [14,15]. Preterm infants have been shown to have higher rates of childhood hospitalization compared to infants born closer to term [16,17].

The primary objective of this study was to characterize the burden of prematurity in Canada over the first ten years of life—as characterized by healthcare resource utilization, direct medical costs, indirect costs associated with lost productivity, and mortality—to describe trends in utilization patterns from infancy and into childhood, and across gestational-age categories. These costs are characterized both as cost per individual preterm infant, and scaled to the Canadian population level by extrapolating individual costs to the number of preterm infants born each year in Canada, and the corresponding gestational age distribution.

## Methods

**Figure 1:**
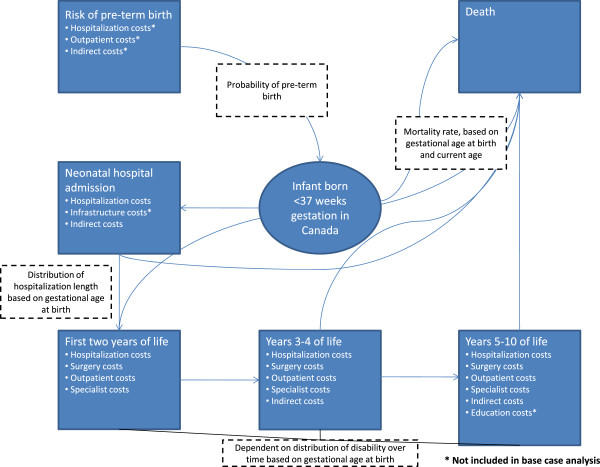
Schematic of Markov model structure for estimating economic burden of prematurity in Canada.

Data from longitudinal, administrative population-based databases from Québec, Canada were used to meet this objective. The methodology presents a Canadian adaptation of a previously developed burden of illness model from the United Kingdom (UK) estimating the long-term costs of preterm birth throughout childhood in England and Wales, [13] based on the incorporation of population-based empirical resource utilization data from Québec. Consistent with other recent studies, [18] we assumed that the population-based Québec data were generalizable to the Canadian population, and the overall economic burden of prematurity in Canada was estimated using a Markov decision model. The model structure is shown in Figure 1. Infants entering the model were stratified by gestational age at birth, with early preterm defined as <28 weeks, moderate preterm defined as 28–32 weeks, and late preterm defined as 33–36 weeks [13]. Costs were included from the time of prenatal care through the first ten years of life for surviving preterm infants. Overall components of costs included in the model were medical costs (for both the infant and excess prenatal costs for the mother) and indirect costs associated with lost productivity for parents. Costs of education, additional prenatal care, and of building neonatal facilities were considered in sensitivity analysis.

### Data source

Resource utilization parameters were populated using Régie de l'assurance maladie du Québec (RAMQ) physician billing data from Québec, Canada linked to MED-ÉCHO hospital discharge abstract databases. A retrospective population-based design was used to establish and follow a birth cohort of all premature infants born during 1996–1997 until age ten. The RAMQ insures all provincial health plan registrants in Québec (99% of 7,731,600 Québec residents in 2006) for necessary medical and hospital services and their databases include: 1) claims [19,20] from the approximately 92% of Québec physicians who work on a fee-for-service basis, [20] and 2) all acute care hospital discharge abstracts in the province. Ethical approval of the protocol and data release was provided by the Commission d’acces à l’information du Québec. The Markov model used to estimate the economic burden of prematurity in Canada, adapted from an alogous model developed for the the UK, was developed in Microsoft^®^ Excel. Data from the RAMQ was stored in a SQL database (Microsoft^®^ SQL version 10.50.1600.1), and analysis was conducted using R 2.13.1.

### Model structure

Epidemiological and resource utilization parameters were stratified by gestational age category, and overall results are a weighted average of gestational age-specific results and cost parameters, based on the relative distributions of early, moderate, and late preterm infants. Following live birth, infants who did not die in the delivery room went on to either admission to a neonatal care facility or discharge directly home. Following hospital discharge, a single model state was used to describe time until age two to account for increased medical costs incurred during early childhood. Following age two, costs were accrued annually until age ten. Level of disability was incorporated in a sensitivity analysis to characterize costs associated with special education requirements. Disabilities included motor function (including cerebral palsy), vision and hearing impairment, and cognitive abilities, consistent with the definitions used within the VICSG cohort [21]. Children were eligible to shift across disability states over time based on a Markov model structure, in which the probability of entering a disability state in a given year was dependent solely on the current disability state. At age two, the distribution across disability levels was based on gestational age at birth; in subsequent years, a Markov transition model was used to describe shifts in disability levels over time [13]. Between ages two and ten, medical costs were accrued annually based on observed resource utilization and costs by gestational age category from the RAMQ data.

The probability of live discharge from the neonatal intensive care unit, by gestational age, were taken from a study by the Canadian NICU Network during 1996–1997 [22]. Additional parameters describing survival probabilities from birth to age ten, and trajectories of disability over time were taken from a published decision model, [13] with the exception of the gestational-age specific probability of death in the delivery room or in the neonatal intensive care unit, which were taken from a more recent publication based on a population-based study of all births in New South Wales and Australian Capital Territory in Australia [23]. The RAMQ data did not contain sufficient information to compute all survival-related parameters for Canada, but where available they were computed and found to be comparable to those calculated for the UK and Australia [23-25].

Costs were discounted at 5% annually.

### Additional costs considered in sensitivity analysis

Several additional costs associated with preterm birth were considered in exploratory sensitivity analysis. The primary analysis did not incorporate these elements because empirical data were not available to calculate the relevant parameters, which were instead estimated based on assumption and expert opinion.

Excess prenatal costs were included in addition to costs associated with the infant following birth. These costs were defined as those associated with additional resource utilization incurred by women identified as high-risk for preterm labor and were based on published sources and expert opinion (Additional file 1: Table S1). In absence of published literature, clinical expert consultation was sought and it was assumed that 50% of preterm births were associated with excess prenatal costs, and the remaining 50% of preterm births were not identified in pregnancy and as such were not associated with excess prenatal resource utilization. Education costs associated with special education requirements for children with disability included from age five onwards. The additional contribution of infrastructure cost to neonatal facility per-diem costs was based on the assumption that a neonatal facility would cost $2.5 million to build, would contain 25 infant-beds, and would have an effective lifetime of 30 years [26,27]. Empirical data were not available for these parameters. This resulted in an additional cost of $18.26 per infant per day associated with neonatal care [28].

### Cost parameters

Model parameters associated with resource utilization, the epidemiology of preterm birth, and Markov model transition probabilities were taken from a published model [13]. National-level costs were based on an assumption of 380,863 live births in Canada, [29] with 0.40% of births early preterm, 1.14% moderate preterm, and 6.19% late preterm [30].

Unit costs were based on Ontario 2012 costs; the most recent available costs were taken from published sources and were inflated as needed to 2012 values as needed using inflation indices based on the Statistics Canada Consumer Price Index (Additional file 1: Table S2). Unit costs used within the model are given in Table 1. The average unit cost of $111.88 associated with in-hospital procedures was calculated by multiplying unit costs from the 2012 Ontario schedule of physician benefits [31] to the ten most commonly listed procedures within the RAMQ data.

**Table 1:** Unit costs used associated with the burden of prematurity in Canada

**Item**	**Unit cost ($CAD)**	**Source/comments**
Prenatal unit costs for women at risk of preterm labor*		
Inpatient days	809.87	Canadian Institute of Health Information [7]
Additional midwife visits	23.20	Working in Canada [32]
Additional obstetrician visits	101.70	OHIP Schedule of benefits [30]
Cervical cerclage	145.10	OHIP Schedule of benefits [30]
Beta agonists	6.00	Ontario Drug Benefit formulary [33]
Oxytocin receptor antagonists	67.75	OHIP Schedule of benefits [30]
Unit costs for different modes of delivery*		
Spontaneous delivery	498.70***	OHIP Schedule of benefits [30]
Instrumental delivery	625.66***	OHIP Schedule of benefits [30]
Elective caesarean	757.11***	OHIP Schedule of benefits [30]
Emergency caesarean	786.51***	OHIP Schedule of benefits [30]
Unit costs associated with neonatal intensive care unit for preterm infants*		
Neonatal intensive care	1,628.60	OCCI [34]
Neonatal normal care	388.00	OCCI [34]
Parameters associated with neonatal intensive care unit infrastructure		
Cost of building a facility	2,500,000	Assumption
Number of infants cared for simultaneously	25	Assumption
Lifetime of facility (years)	30	Assumption
Resulting cost per infant per day	9.13	Function of above parameters
Unit costs between hospital discharge and age 2 years for preterm infants		
Inpatient stay (per day)	628.49	Harris 2011 [35]
Outpatient visits	NA	Physician billings per visit used directly from RAMQ
Unit costs incurred between age 2 and 10 years for preterm infants*		
Pediatric ICU stay (per day)	2,002.86***	Harris 2011 [35]
Pediatric procedures	111.88*	RAMQ and OCCI [34]
Other pediatric inpatient stay (per day)	628.49	Harris 2011 [35]
Outpatient visits	NA	Physician billings per visit used directly from RAMQ
Mainstream primary school	7,720.05^*^	Learning Disabilities in Canada Report [12]
Special school	15,666.45^*^	Learning Disabilities in Canada Report [12]
Indirect costs incurred by families of preterm infants		
Gross pay per hour (male, full-time)	26.33	Statistics Canada (June 2012) [36]
Gross pay per hour (female, full-time)	23.18	Statistics Canada (June 2012) [36]
Gross pay per hour (male, part-time)	15.62	Statistics Canada (June 2012) [36]
Gross pay per hour (female, part-time)	17.36	Statistics Canada (June 2012) [36]

*Costs were inflated to 2012 where appropriate by using Canadian health inflators; ***Weighted average inflated to 2012; CIHI = Canadian Institute for Health Information; OCCI = Ontario Case Costing Initiative; OHIP = Ontario Health Insurance Plan; RAMQ = Regie de l'assurance Maladie du Quebec.

Indirect costs associated with parental time taken off of work to attend any medical visits and hospitalizations incurred by their child were included. It was assumed that these costs would be incurred from ages two onwards, and that premature infants would have a full-time caregiver available for medical appointments and hospitalizations from discharge until age two. From age two onward, indirect costs due to lost productivity were calculated, stratified by gestational age category, based on the number of outpatient visits and inpatient days observed in the RAMQ database. It was assumed that outpatient visits would be associated with two hours taken off work and that inpatient days would be associated with eight hours taken off work, and assumed an hourly wage of $23.18, based on a full-time female employee.

### Resource utilization parameters

The linked RAMQ and MED-ÉCHO databases were used to extract the following resource utilization and cost parameters, stratified by gestational age category and current age: number of hospital days, stratified into general ward and intensive care unit; surgeries and other procedures received in hospital; and outpatient costs billed by the physician. Data were extracted from 1996 to 2007 inclusive, for all preterm infants born in 1996 and 1997. Infants were excluded from the analysis if no subsequent medical or hospital visits occurred after the initial birth hospitalization and no record of death could be found. Infants were also excluded if a transfer to another hospital during the initial birth hospitalization was recorded, due to inconsistencies in the data associated with these entries. In estimating cumulative costs throughout childhood within the decision model, age-specific costs for each gestational age category were weighted by the proportion that an infant would survive to that age. The percentage distribution of utilization of mainstream primary education and special education by disability level, considered in sensitivity analysis, is given in Additional file 1: Table S3.

### Canadian resource utilization

Average resource utilization and costs per child were extrapolated to Canadian estimates by multiplying costs by the estimated number of live births [29] and the proportion of premature births in Canada [30]—both overall prematurity and stratified by gestational age category.

### Probabilistic sensitivity analysis

A probabilistic sensitivity analysis (PSA) was undertaken to assess the impact of uncertainty in model input parameters on potential variability of overall total cost results. The epidemiological parameters that were assumed to be consistent with those reported previously were assumed to follow the described distributions [25]. For *de novo* Canadian resource utilization and cost parameters, standard errors were estimated directly from the RAMQ data, and normal distributions were assumed.

## Results

**Figure 2:**
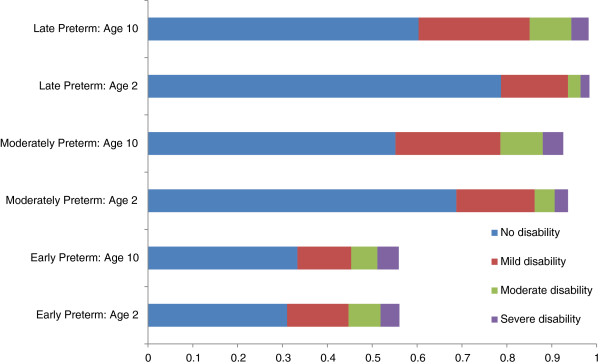
Distribution of Canadian live births across disability levels for preterm infants by gestational age.

The distributions of survival and disability at ages two and ten, respectively, stratified by gestational age at birth, are shown in Figure 2. Based on clinical input parameters, model projections estimated that the survival rate amongst live births at age two would be 56.0% of early preterm infants, 92.8% of moderate preterm infants, and 98.4% of late preterm infants. The corresponding survival rates at age ten were 55.9% for early preterm, 92.6% for moderate preterm, and 98.2% for late preterm, reflecting the small mortality rates between age two and age ten for all gestational age categories. Compared to survival, there was greater variation in distribution of disability between age two and age ten. For moderate and late preterm babies there was a shift from no and mild disability to moderate and severe disability, although the majority of children remained in the no disability state at age ten. For early preterm babies, the relative proportion of severe disability was greater at age two, with a small shift from moderate and severe disability to no disability and mild disability at age ten.

**Figure 3:**
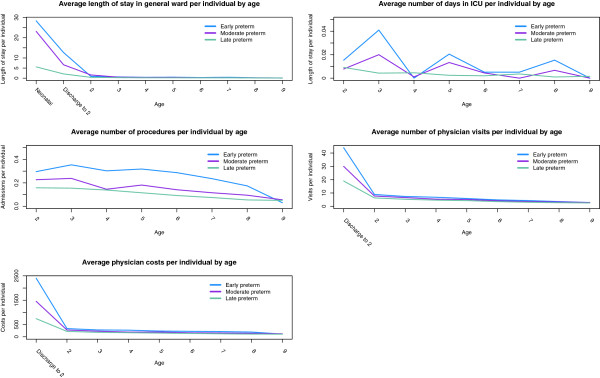
Resource use per individual in the Québec cohort from birth to age ten.

Figure 3 describes resource utilization (hospitalizations, hospital days, inpatient interventions, intensive care unit visits, and outpatient physician visits) from birth to age ten, separated by gestational age category. Total inpatient days and outpatient costs are reported in Table 2. All resource utilization notably decreased with age across all gestational age categories. For moderately preterm infants, hospital days ranged from 1.6 days at age two to 0.09 days at age ten. Costs associated with outpatient visits for moderately preterm infants ranged from $1,453 prior to age two to $123 at age ten. Resource utilization tended to be similar between early and moderate preterm infants, and higher for these categories compared to late preterm infants. In these analyses, results at each age are specific to the subset of individuals who survived until that age, i.e. for any given age group, all resource utilization analyses were based on a denominator of surviving infants, and infants who died prior to that age were excluded from analysis. When calculating resource utilization from the RAMQ database, it was assumed that early and moderate preterm infants with no record of neonatal hospitalization died prior to admission or were otherwise lost to follow up and were excluded, while late preterm infants with no record of neonatal hospitalization were assumed to have been discharged directly home.

**Table 2:** Average number and associated standard error of inpatient hospital days, outpatient costs, and associated indirect costs incurred due to lost productivity by caregivers of preterm infants from age 2–10, stratified by gestational age at birth and current age

	**Early preterm infants (<28 weeks)**			
	**Inpatient days**		**Outpatient costs**	
	**Mean**	**Standard error**	**Mean**	**Standard error**
Discharge-Age 2	17.45	3.32	2403.02	223.01
Age 2	0.89	0.21	336.17	24.82
Age 3	0.65	0.20	280.46	22.61
Age 4	0.42	0.13	271.14	32.03
Age 5	0.50	0.19	234.18	25.90
Age 6	0.32	0.09	223.13	26.59
Age 7	0.45	0.20	212.22	27.18
Age 8	0.24	0.08	193.54	28.88
Age 9	0.04	0.02	110.47	9.07
	**Moderately preterm infants (28–32 weeks)**			
	**Inpatient days**		**Outpatient costs**	
	**Mean**	**Standard error**	**Mean**	**Standard error**
Discharge-Age 2	8.75	1.27	1453.34	102.99
Age 2	1.60	0.80	277.79	16.29
Age 3	0.50	0.13	230.30	14.57
Age 4	0.35	0.08	191.50	8.69
Age 5	0.39	0.09	195.20	8.35
Age 6	0.24	0.04	163.56	7.65
Age 7	0.22	0.06	155.44	10.27
Age 8	0.19	0.05	142.84	9.50
Age 9	0.09	0.02	123.19	6.80
	**Late preterm infants (33–36 weeks)**			
	**Inpatient days**		**Outpatient costs**	
	**Mean**	**Standard error**	**Mean**	**Standard error**
Discharge-Age 2	2.40	0.17	734.14	12.87
Age 2	0.40	0.04	220.42	220.42
Age 3	0.26	0.02	182.21	182.21
Age 4	0.20	0.01	165.21	165.21
Age 5	0.16	0.01	154.99	154.99
Age 6	0.20	0.06	133.59	133.59
Age 7	0.17	0.03	122.36	122.36
Age 8	0.10	0.01	109.91	109.91
Age 9	0.10	0.02	107.13	107.13

The total economic burden of prematurity by category of expenditure (neonatal costs,direct medical costs in subsequent years, and lost productivity costs) is reported in Table 3, both aggregated over all preterm infants and stratified by gestational age at birth. Conversely to the above-described analyses in Figure 3, costs per infant were averaged over all live births, including those who died during infancy or childhood, and costs throughout childhood are downweighted as applicable to reflect the smaller surviving population size at each year of life. Cost per infant over the first ten years of life was estimated to be $67,467 (PSA 2.5th-97.5th percentiles: $52,796-$83,206) for early preterm infants, $54,554 (PSA 2.5th-97.5th percentiles: $46,301-$66,422) for moderate preterm infants, and $10,010 (PSA 2.5th-97.5th percentiles: $ 8,649-$13,296) for late preterm infants. Based on population sizes this corresponds to total national costs of $123.3 million for early preterm infants, $255.6 million for moderate preterm infants, $208.2 million for late preterm infants, and $587.1 million for all infants. While individual-costs per infant were highest for moderately preterm infants, national-level costs were greater for moderate and late preterm infants due to the larger population size.

**Table 3:** Individual and national economic burden of prematurity in Canada ($CAD), stratified by gestational age

	**Gestational age at birth**							
	**All preterm infants (<37 weeks)**		**Early preterm (<28 weeks)**		**Moderately preterm (28–32 weeks)**		**Late preterm (33–36 weeks)**	
	**n = 27,308**		**n = 1,828**		**n = 4,685**		**n = 20,795**	
	**Total cost ($1,000,000)**	**Cost per infant ($)**	**Total cost ($1,000,000)**	**Cost per infant ($)**	**Total cost ($1,000,000)**	**Cost per infant ($)**	**Total cost ($1,000,000)**	**Cost per infant ($)**
Delivery costs	16,722,919	612	1,057,377	578	2,920,146	623	12,745,395	613
Neonatal costs	374,121,486	13,700	97,833,950	53,520	198,164,090	42,298	78,123,447	3,757
Costs discharge to age two	94,081,058	3,445	16,963,439	9,280	30,793,148	6,573	46,324,421	2,228
Costs incurred ages two-four								
Medical	32,694,477	1,197	2,511,003	1,374	9,070,596	1,936	21,112,878	1,015
Indirect	23,662,266	866	1,599,934	875	5,322,006	1,136	16,740,327	805
Costs incurred ages five to ten								
Medical	23,901,819	875	1,934,994	1,059	5,044,290	1,077	16,922,535	814
sIndirect	21,899,601	802	1,429,570	782	4,272,425	912	16,197,606	779
Total costs	587,083,627	21,498	123,330,267	67,467	255,586,702	54,554	208,166,658	10,010
Total costs: PSA* 2.5th percentile	507,206,197	18,754	96,510,494	52,796	216,918,336	46,301	179,848,879	8,649
Total costs: PSA* 97.5th percentile	732,354,145	26,818	152,099,814	83,206	311,185,320	66,422	276,500,332	13,296
Total costs: Including prenatal costs, neonatal infrastructure, and special education	2,430,359,101	88,988	216,584,016	118,481	576,661,757	123,087	1,637,113,329	78,726

*PSA = Probabilistic sensitivity analysis.

At the individual level, for all gestational age categories, the largest contributor to total costs was the cost associated with the neonatal intensive care unit stay, followed by medical costs incurred between discharge and age two. Across categories of expenditure, individual-level costs tended to be highest for early preterm infants prior to age two, followed by moderate preterm infants and late preterm infants. Prior to age two, costs were similar between moderate and preterm infants, and substantially lower for late preterm infants. The most substantial cost differences were in neonatal hospitalization which ranged from $3,768 in late preterm infants to $53,308 in early preterm infants. After age two, costs were comparable across all age categories, although this implies that costs incurred by surviving children were highest for early and moderate preterms as the denominator was all live births, and there was notably higher mortality following live births for the earlier gestational age categories.

## Discussion

In this study, a decision model was used to capture trends in survival, resource utilization, and indirect costs over the first ten years of life for preterm infants in Canada. A rigorous and comprehensive decision model, originally developed for the UK, was adapted to the Canadian setting by updating unit costs to Canadian values, and quantifying resource utilization by age and gestational age at birth category using a population-based real-world administrative data source. The results of this study allow for potential interventions to delay or prevent preterm birth, or to prevent morbidity in preterm infants to be contextualized with respect to the overall burden.

A recent study published by Landry et al. reported resource utilization for infants born in Québec from 1983–1992, although this was restricted to infants with respiratory complications, and utilization and costs were not further stratified by gestational age at birth [18]. The study describes here includes resource utilization and associated costs for all preterm infants born during 1996–1997, regardless of specific complications, and all results are stratified by gestational age at birth. Total medical costs were higher in the Landry et al. study, $10,719-$13,472 per person-year across respiratory distress syndrome (RDS) and bronchopulmonary dysplasia (BPD) complications, respectively, compared to $22,794 over ten years estimated hear as a weighted average of direct medical costs and lost productivity costs over the first ten years of life. This discrepancy is explained in part by the restriction in the Landry et al. study to infants with RDS and/or BPD, who would be expected to incur greater resource utilization and costs as a result of these co-morbidities. In addition, in the study reported here, the denominator was all live births, such that infants who died during the ten-year follow-up period would only contribute resource utilization and costs until time of death; this is particularly notable for extremely preterm infants, for whom over 40% were estimated to die prior to age two. While Landry et al. considered pharmaceutical costs, and this study did not, this is not anticipated to be a major source of discrepancy as they comprised a relatively minor proportion of overall medical costs (approximately 1-2%) [18].

Consistent with the medical literature, a dramatic improvement in survival in moderate and late preterm infants relative to early preterm infants was observed. Not surprisingly, neonatal intensive care costs were the largest contributor to overall medical costs amongst all preterm infants. Neonatal costs associated with moderate preterm infants were found to be similar to early preterm infants. When considered in exploratory sensitivity analysis, education costs were an important cost driver, and were highest in late preterm infants, due to the larger number of survivors and the larger proportion attending a mainstream primary school, relative to earlier preterm categories with higher prevalence of severe disability, associated with not attending a mainstream school (Additional file 1: Table S3).

The general trend in overall costs was that early and moderate preterm infants tended to incur similar costs, with much lower costs observed in late preterm infants. Based on model structure, there are two main determinants of costs incurred: the proportion surviving and the level of resource utilization incurred by survivors. Amongst surviving infants, early preterm infants tended to have the greatest medical resource utilization amongst all categories of utilization and at all ages (Figure 3). However, these infants also experienced the lowest survival rates, such that a smaller proportion of live-born preterm infants survive into childhood and incur related costs (Figure 2). Thus, the similar cost per infant for moderate preterm infants relative to early preterm infants is reflective of the higher survival rate in moderate preterm infants which results in a greater proportion of infants incurring costs throughout childhood.

A key strength of this study is the high quality and comprehensive nature of the data used to populate model parameters. The RAMQ data describe population-based resource utilization for all preterm infants born in the province of Québec during 1996 and 1997 over their first ten years of life. These resource utilization data were combined with a published model describing the epidemiology, survival, and disability trajectories of preterm infants, and unit costs for health resources were updated to 2012 values using inflation factors. Thus, the Canadian adaptation of the model provides an up to date and comprehensive estimate of the overall economic burden of prematurity in Canada. In addition, these results are potentially generalizable beyond Canada to countries with similar trends in pediatric treatment patterns and relative costs of health resources.

Limitations to the approach include the fact that prescription medication costs were excluded from the analysis as the RAMQ data only include prescription records for a subset of individuals with medication coverage. The assumption was made that for preterm infants during childhood, the costs associated with medications would be substantially less important than those associated with hospitalizations and outpatient visits. In addition, in order to maximize length of available follow-up, the analysis was based on a cohort of infants born during 1996–1997, and, as such, their patterns of care may not reflect current treatment practices. Results were scaled to the population of Canada based on the assumption that clinical outcomes and resource utilization in Québec, and unit costs for medical resources from Ontario would be generalizable to the rest of Canada. While health care in Canada is delivered at the provincial level, it was assumed that individual provinces would not vary substantially with respect to pediatric clinical outcomes, resource utilization, and unit costs, such that the observed values for Québec and Ontario could serve as a suitable approximation for other provinces. Ontario, the largest Canadian province was selected as the most applicable province for selecting unit costs. While model parameters associated with survival and long-term disability trajectory were taken from UK and Australian sources where not available for Canada, where values were available for multiple sources (e.g. probabilities of live discharge from hospital for gestational age 23–35 weeks were available for both Canada [22] and the UK, [25]while probabilities of death in the delivery room for gestational ages 23–31 weeks were available for both Australia [23] and the UK [25]), they were compared and found to be comparable, supporting the generalizability of such parameters across health care systems. This is further supported by an international comparison of perinatal and infant mortality statistics, in which similar results were reported for Canada, the UK, and Australia (Tables 2–4 of reference) [24].

The strength of an economic model is dictated by the strength of evidence used as model inputs. The primary, core analysis was based on inputs for which empirical data were available, including the incidence of premature birth and gestational age distribution in Canada, and survival and resource utilization and costs associated with preterm infants in Québec. While the highest quality evidence available was used in the primary analysis and sensitivity analyses, where empirical evidence was lacking, expert opinion evidence was used as sensitivity analysis, for prenatal resource utilization, special education associated with disability, and construction of neonatal facilities. For the inclusion of excess healthcare utilization for prenatal care, it was assumed that 50% of preterm births would have been associated with such care, due to a paucity of published estimates. In adapting the model to a Canadian setting, it was assumed that the values assumed within the UK model describing distribution of disability, and requirements for special needs education would be relevant for Canada.

Future extensions of this work include the assessment of temporal trends in care to project expected updates to utilization estimates, and to compare the costs of preterm infants to those incurred by full-term infants in order to estimate an incremental cost of prematurity in addition to the absolute costs presented here. In addition, it would be of interest to expand the burden of illness model to compare differences in economic burden with respect to specific medical conditions relative to prematurity and pediatric populations, such as respiratory morbidity. Finally, the incorporation of quality-of-life estimation and empirical estimation of out-of-pocket expenses and lost productivity costs in Canadian families in addition to survival, resource utilization, and economic outcomes could provide a more inclusive view of the burden of prematurity throughout childhood, and would allow for a more comprehensive comparison of the overall burden experienced during childhood by preterm infants born at varying gestational ages. The model described here allows for numerous “what if” scenarios to be considered in future consideration of additional research questions.

## Conclusion

Premature birth results in significant infant morbidity, mortality, healthcare utilization and costs in Canada. The results of this study allow for potential interventions to delay or prevent preterm birth, or to prevent morbidity in preterm infants to be contextualized with respect to the overall burden. A comprehensive decision-model based on analysis of a Canadian population-based Canadian administrative data source suggested that substantial costs per infant are observed in early and moderate preterm infants, but when scaled to the national level, late preterm infants contribute a substantial burden due to the relatively larger population size. Although the highest medical costs are incurred during the neonatal period, higher resource utilization and costs extend into childhood.

## Competing interests

P. Vo is an AbbVie Inc employee and may hold stock or options in AbbVie Inc.

K. Gooch is an AbbVie Inc employee and may hold stock or options in AbbVie Inc.

This study was funded by AbbVie, North Chicago, IL.

## Authors’ contributions

KG developed study objectives and reviewed and provided significant feedback on the manuscript. KMJ designed and built the model, and wrote the first draft of the manuscript. EK performed statistical analysis of RAMQ data. EO contributed to literature review, and manuscript writing. AL and PV provided significant feedback on the manuscript. All authors read and approved the final manuscript.

## Pre-publication history

The pre-publication history for this paper can be accessed here:

http://www.biomedcentral.com/1471-2431/14/93/prepub

## Supplementary Material

Additional file 1 Supplementary data input tables. Click here for file
