# Supplementary material for: The economic burden of prematurity in Canada
Source: BMC Pediatr. 2014 Apr 5;14:93. doi: 10.1186/1471-2431-14-93 (PMC4108009; doi:10.1186/1471-2431-14-93)
Supplement: Additional file 1 — Supplementary data input tables. [file 1471-2431-14-93-S1.docx]

**Additional file 1 Supplementary data input tables**

Additional file 1: Table S1: Excess prenatal resource utilization associated with women identified as high risk for preterm labour.

|  | Gestational age at birth | | | Source |
| --- | --- | --- | --- | --- |
|  | Early preterm infants (<28 weeks) | Moderately preterm infants (28-32 weeks) | Late preterm infants (33-36 weeks) |  |
| Additional inpatient days | 14 days | | | Expert Opinion (Dr. Pamela Bradt, personal communication) |
| Additional obstetrician visits | 12 visits | | | Expert Opinion (Dr. Pamela Bradt, personal communication) |
| Cervical cerclage | 1% | | | Vidaeff AC, Ramin SM: Management strategies for the prevention of preterm birth: Part II - Update on cervical cerclage. *Curr Opin Obstet Gynecol* 2009, **21:**485–490. |

Additional file 1: Table S2: Price inflation factors indices to update costs from 2005 to 2012 $CAD

| Year | Statistics Canada Consumer Price Index (2002=100) |
| --- | --- |
| 2005 | 106.4 |
| 2006 | 108.7 |
| 2007 | 110.8 |
| 2008 | 112.8 |
| 2009 | 115.9 |
| 2010 | 118.5 |
| 2011 | 119.8 |
| 2012 | 120.4 |

Additional file 1: Table S3: Education utilization from ages five – ten by disability status

| Year | Mainstream primary school (%) | Special education (%) |
| --- | --- | --- |
| No disability | 100 | 23 |
| Mild disability | 100 | 34 |
| Moderate disability | 93 | 64 |
| Severe disability | 42 | 80 |
